# Impact of immobilization system angle, body mass index and breast size on breast radiotherapy accuracy using EPID-only setup

**DOI:** 10.1016/j.heliyon.2025.e42176

**Published:** 2025-01-22

**Authors:** Ioana-Claudia Costin, Loredana G. Marcu

**Affiliations:** a West University of Timisoara, Faculty of Physics, 300223, Timisoara, Romania; b Bihor County Emergency Clinical Hospital, Oradea, 410167, Romania; c Faculty of Informatics & Science, University of Oradea, Oradea, 410087, Romania; d UniSA Allied Health & Human Performance, University of South Australia, Adelaide, SA, 5001, Australia

**Keywords:** Breast cancer, IMRT, VMAT, Patient setup, Systematic error, Random error

## Abstract

This study aims to assess setup errors for patients immobilized on different board inclinations and to evaluate the effect of body mass index (BMI) and breast size on positioning errors. Furthermore, the dosimetric impact of setup errors on target and organs at risk was measured using three different irradiation techniques (3D conformal radiotherapy, intensity modulated and volumetric modulated arc radiotherapy). A cohort of 40 breast cancer patients was split into two groups as a function of immobilization board inclination: 20 patients immobilized on 7.5° inclination (group A) and 20 on 0° (group B). Systematic and random errors were determined with weekly portal and tangential images. A strong correlation between BMI and both systematic (r = 0.720) and random errors (r = 0.752) was observed in posterior direction for group B, while the correlation between breast size and setup errors showed a moderate association under systematic errors for right (r = −0.507) and left (r = 0.503) directions. The dosimetric impact of setup errors on target volume showed higher contribution from systematic than from random errors. Suboptimal coverage of target volume was more prominent in group A for all planning techniques (46.65Gy 3DCRT, 46.95Gy IMRT, 46.90Gy VMAT). Patients with high BMI could benefit from the inclined immobilization board with a higher frequency of image position verification. When comparing 3DCRT versus modulated techniques the ipsilateral lung is better spared with the latter, while the contralateral lung is more efficiently protected with conformal technique.

## Highlights

• Setup errors are higher on inclined board versus flat plane.
• Patients with high BMI showed higher setup errors in posterior direction.
• Larger breast size induces higher setup errors.
• Lung sparing better in modulated intensity techniques.
• Modulated techniques more robust to setup errors than conventional technique.

## Graphical abstract

**Figure:**
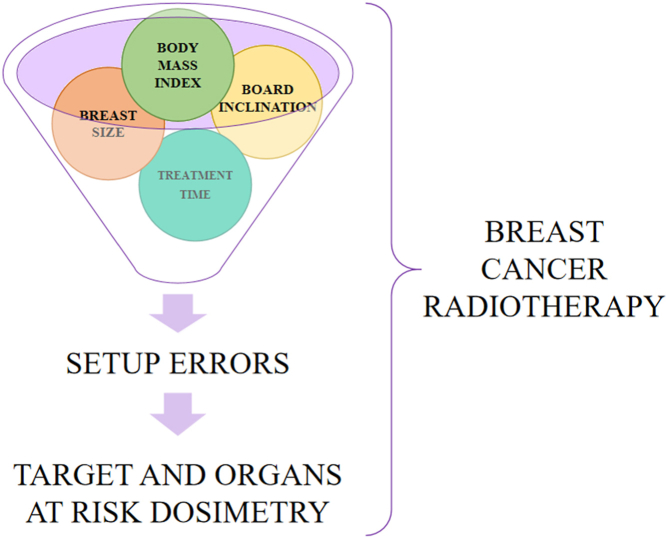

## 1 Introduction

Breast cancer is one of the most common types of cancer among women worldwide [1]. Radiation therapy is used for the treatment of breast malignancies in all cancer stages, both with the aim to eradicate the tumor and to reduce the risk of recurrence after surgery. Current radiotherapy techniques employed for breast cancer management include conformal therapy (3D conformal radiotherapy - 3DCRT) and modulated techniques (intensity modulated radiotherapy - IMRT and volumetric modulated arc radiotherapy - VMAT). While brachytherapy is also a treatment option for breast cancer patients using the MammoSite balloon or I-125 seed implants, intensity modulated proton therapy is gaining more interest owing to the superior physical and radiobiological properties of protons vs photons and to the increasing number of proton facilities throughout the world [2,3].

To achieve high survival rates among breast cancer patients, the efficiency and accuracy of radiotherapy delivery are critical factors, both in terms of tumor control and dose received by healthy tissues. Given that radiotherapy is administered in a fractionated manner, accurate patient setup before each fraction as well as reproducibility of patient positioning are key factors to be considered for a successful treatment delivery.

Breast cancer radiotherapy involves the use of immobilization boards to limit patients' movement during the course of therapy and to reproduce patient positioning. Systematic and random errors are the main setup errors evaluated for treatment delivery accuracy. Systematic errors (Σ) are mainly attributed to issues related to the initial position adjustment, representing the difference between the patient's position at the CT simulator and the treatment position verification image. Random errors (σ) are attributed to day-to-day variations in patient positioning [4]. Uncorrected setup errors can have further implications, affecting treatment delivery accuracy and the dosimetric outcome.

The use of board immobilization is an important aspect of treatment personalization and accurate treatment delivery. Among all the immobilization techniques, the most widespread are those adjusted for patient's anatomy and comfort. Recent studies evaluated the difference between vacuum mattresses of various sizes [5] and different hand-holder techniques [6] for reducing setup errors and optimize treatment outcome. Studies on vacuum mattresses for immobilization concluded that large sided and long mattresses ensure superior fixation of the chest wall, head and limbs as compared to smaller ones which also induce larger random errors [5]. Skin tattoos, either with black ink or ultraviolet ink are a handy tool for patient setup and repositioning, although accurate reproducibility of patient positioning is often a challenge with this technique. Their shortcomings include skin mobility, misidentification, and difficulty in their perceptibility when dark color tattoos are used [7,8]. An efficient replacement for tattoos is surface guided radiotherapy (SGRT), an optical surface tracking system that is able to evaluate the patient's surface anatomy, being sensitive to variations in contour. Studies reported notable reduction in setup errors when tattoo-based positioning was replaced with SGRT [9], while others have emphasized the role of surface guided systems to protect the organs at risk (OARs) using deep inspiration breath hold technique (DIBH) [10,11].

Beside the immobilization system, another aspect evaluated by some studies is the position of the patient during the radiotherapy sessions, whether supine, prone or lateral. Concerning the frequency of position verification images, different protocols were reported in the literature (no image guidance, weekly image guidance, first 3 days of treatment followed by weekly imaging, etc.) to assess systematic and random errors for prone position relative to the chest wall. The frequency of image verification was shown to be an important factor in diminishing setup errors, while the latter protocol mentioned above appeared to reduce both systematic and random errors [12]. Regarding organs at risk sparing, Varga et al. evaluated two groups of patients, one supine and one prone, showing a dosimetric advantage for lung doses in the prone position compared to the dose received in supine position; however, for the heart, the dose reduction was minimal [13].

Despite the broad availability of various immobilization devices and of imaging systems for pre-treatment positioning verifications, there are still several middle- and low-income countries where the more classical approaches towards treatment optimization are prevailing. Therefore, it is important to evaluate positioning errors as well as their correlations with patient- and treatment-related parameters with the available devices at one's hospital in order to improve patients' outcome. In view of this, our study aimed to evaluate the influence of the immobilization board's tilt on setup errors, including as a variable the patient's habitus and treatment-related factors. While a number of studies analyzed the correlation between body habitus and setup errors, most of them used different immobilization systems from ours (vacuum bag, baseplate, custom-made systems) and were assessed mostly with cone beam CT [[14], [15], [16], [17], [18]]. Our study employed EPID-based position verification according to the internal protocol due to lack of 3D imaging techniques for patient setup verification. The study is a single-institutional study, following the internal protocol for patient position verification (portal and tangential images) in breast cancer radiotherapy using two immobilization board settings (no plan inclination versus plan inclination) with the patient in supine position. Finding correlations between positioning errors and patient-related factors (such as body weight and tumor volume) or treatment-related factors (board angulation, treatment time and radiotherapy delivery technique) can offer valuable information on dosimetric aspects related to the tumor and OARs.

In view of the above, the purpose of our study is to bring to the fore a key aspect of radiotherapy - treatment optimization through the evaluation of setup error occurrence, their correlation with patient-related factors as well as the influence of setup errors on dosimetric outcome. To fulfil these multiple goals, the following aspects were analyzed and reported in the current study.

(1) evaluation of positioning errors when immobilization boards are used (with and without inclination);
(2) the effect of patient-related factors on setup errors;
(3) the effect of treatment-related factors on setup errors;
(4) dosimetric implications of uncorrected setup errors for the three most commonly employed external beam radiotherapy techniques: 3DCRT, IMRT and VMAT.

## 2 Materials and methods

### 2.1 Patient selection

The study enrolled 40 female patients with confirmed breast cancer who were treated at our department during 2021–2022 with 3D conformal radiotherapy (3DCRT). Fifteen patients presented with right-sided breast cancer while twenty-five patients were treated for left-sided breast cancer. Patients were included in the study regardless of age, tumor stage (from T_1_ to T_4_) and tumor volume (right or left breast, presence/absence of positive lymph nodes and tumor bed). However, the following criteria were imposed for patient selection: they must not have unhealed tissue, bilateral breast irradiation and different fractionation regimes (different than 2Gy/fraction).

Patients’ body mass index (BMI) was calculated, resulting in a mean value of 29.43 kg/m^2^ (ranging from 22.50 kg/m^2^ to 39.70 kg/m^2^) and a median of 27.95 kg/m^2^, while breast size (contoured on treatment planning system) ranged from 255.34 cm^3^ to 3000.97 cm^3^ (mean 1135.29 cm^3^).

All treatment-related methods were carried out in accordance with relevant guidelines and regulations. All experimental protocols were approved by the Bihor County Emergency Clinical Hospital. Informed consent was waived because the research did not enroll patients for the said study. This work analyzed the data of patients that have been treated as per the normal medical protocol without any change induced by this study.

### 2.2 Computed tomography simulation

**Fig. 1:**
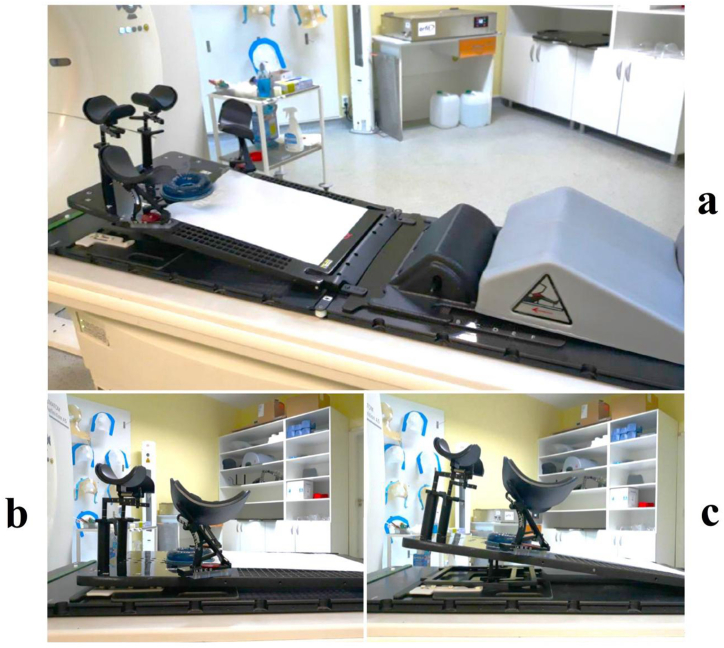
**a.** Quest breast board with bottom stopper and knee holder set on CT simulator couch; **b.** Quest breast board no inclination (Group B positioned on 0° inclination); **c.** Quest breast board 7.5° inclination (Group A positioned on 7.5° inclination).

Computed tomography (CT) simulation was performed with patients in supine position on immobilization boards, marking the isocenter between the last two ribs. The immobilization system used for patient setup was a Quest breast board (Fig. 1a–c) which allowed different inclination of the patient, hand and arm holders, a bottom stopper and knee support (QFix, USA). Patients’ data from CT simulation were manually recorded in Mosaiq (Elekta, Sweden) as follows: hand cup height, arms height, the position of head in the immobilization support, table height (cm), the distance from the mandible to the jugular cavity (cm) and board inclination.

Patients were divided into two groups according to the inclination of the board: group A consisting of 20 patients immobilized on 7.5° inclination and group B with 20 patients immobilized on 0° inclination (no inclination). The 7.5° patient tilt was employed to achieve a parallel position between the sternum and the treatment couch in order to reduce the irradiated volume of ipsilateral lung and heart included in the anterior and posterior tangent. Also, since the gantry of the CT simulator has an aperture of 70 cm, owing to patient-related factors (anatomy or comorbidities) the immobilization on higher inclination (>10°) was not feasible.

### 2.3 Evaluation of patient positioning and treatment time

Patient position was evaluated with weekly orthogonal and tangential portal images (2 pre-session image acquisitions - before and after the shifts) to assess the systematic (Σ) and random (σ) errors for standard coordinates (x, y and z) and also for the following tangential image parameters.

• central lung distance (CLD) which was measured from the posterior limit of the treatment field to the ipsilateral lung contour,
• central beam edge to skin distance (CBESD) measured from the skin to the anterior limit of the treatment field and
• central caudal distance (CCD) measured from the caudal border of the breast to the caudal edge of the treatment field [19].

**Fig. 2:**
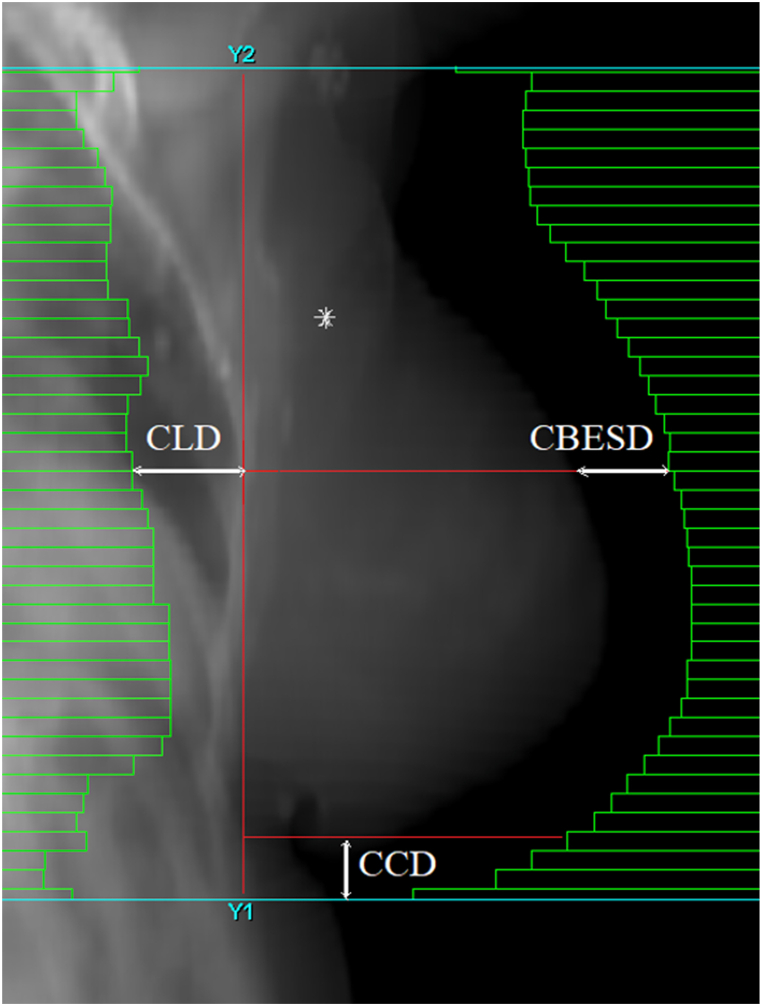
Tangential parameters evaluated in the Monaco 6.1.2 treatment planning system. The image is a digitally reconstructed radiograph showing the following tangential image parameters: CLD = central lung distance, CCD = central caudal distance, CBESD = central beam to edge skin distance.

The measurements were carried out in the treatment planning system (TPS) using the isocenter axis of the anterior tangential field (Fig. 2).

Before each radiotherapy session, patients were positioned based on tattoos previously marked at the CT simulator. The fusion between portal images and digitally reconstructed radiographs (DRR) transferred from the TPS was performed by using internal protocol landmarks to determine the correct patient position. Anterior and lateral images were used for shift determination. Shifts along x (left/right) and y (superior/inferior) axes were assessed from anterior images, while z shifts (anterior/posterior) were determined from lateral images. The landmarks used for position verification were the chest wall, spine and sternum for lateral images, and the ribs, carina and spine for anterior images. The tangential image parameters (CLD, CCD and CBESD) were evaluated using a treatment field image acquisition (the anterior tangent used in treatment planning).

The systematic and random errors were calculated according to van Herk's method [20]. Systematic errors were defined as standard deviation (SD) from mean of the mean individual patient shifts and random errors were evaluated as root mean square (RMS) of SD from the mean individual patient shift.

All errors were assessed according to the internal protocol applied to positioning errors with a range of interest between −2 and +2 mm. Other evaluated intervals were added to strengthen the statement provided by the reference interval (−2 to +2 mm).

Time spent by the patient in a treatment session was evaluated from "beam on" to the end of the treatment fields delivery without patient position verification (treatment time without EPID). In addition, the time spent acquisitioning the portal imagines was also recorded, including the time during which the portals were evaluated (treatment time with EPID verification).

### 2.4 Target and organs at risk definition

The clinical target volume (CTV) consisted of the breast, lymph nodes and boost (labelled as IB = integrated boost). Our department standard margin for planning target volume (PTV) creation is by adding 5 mm in all directions, except the anterior (the target was retracted 3 mm from patient external contour) to the CTV [21]. Setup errors identification and processing for the applied PTV margins was carried out in order for the CTV to receive 95 % of the prescribed dose under setup error influence and to ensure an efficient and personalized treatment.

Target volume and organs at risk were outlined on Monaco TPS by the attending physician in accordance with the international guidelines of the Radiation Therapy Oncology Group (RTOG) [21]. The organs at risk evaluated in this study were the following: heart, ipsilateral and contralateral lung and ipsilateral humeral head.

To determine the margins to be added to the CTV for robustness, three formulas were employed using the following sources: equation (1) van Herk et al. [20], equation (2) Stroom et al. [22] and equation (3) the International Commission on Radiation Units and measurements (ICRU) report No. 62 [23]:

(1) M=2.5Σ+0.7σ

(2) M=2Σ+0.7σ

(3) $$M=\sqrt{\Sigma^{2}+\sigma^{2}}\text{,}$$

where M represents the margins calculated using systematic (Σ) and random errors (σ).

The margins for organs at risk (planning organ at risk volume = PRV) were also evaluated as a function of setup errors owing to the fact that random errors cause blurring in dose distribution, while the systematic errors shift the dose distribution [24]. The formula used for PRV calculation was reported by McKenzie et al. [24]:

$$M_{\text{PRV}}=1.3\Sigma +/-0.5\sigma \text{,}$$

where M_PRV_ represents the margins calculated for OARs, (+) is used when the plateau dose is near OARs meaning that the organ is within the low-dose regions and (−) is used when the dose moves past the organ, into the high-dose regions. The calculation was performed extracting the margin for blurring (-0.5σ) for all organs at risk [24].

### 2.5 Treatment planning

All patients were treated with 3D conformal radiotherapy in normal breathing using the Elekta Synergy Platform. Plans were also simulated for the intensity modulated techniques for each patient in order to evaluate the target coverage (whole breast, integrated boost coverage and hotspot) and organs at risk (OARs).

The prescribed dose for the target volume (CTV) was 50 Gy in 25 fractions and 10 Gy in 25 fractions for the integrated boost (the equivalent dose of 66 Gy delivered in 2 Gy/fraction) [21].

The dosimetric evaluation of the organs at risk and target volume was performed by simulating the setup errors (systematic and random errors) on Monaco TPS by modifying the initial treatment plan isocenter according to systematic and random errors, calculating the isodoses distribution and comparing, before and after, the quantified values provided by dose-volume histograms (DVH).

Dosimetric indices of interest in healthy tissue consisted of the mean dose (D_mean_) of heart and lungs, along with maximum dose (D_max_) and volume percentages (V_5_, V_20_ and V_25_). For CTV, the dose delivered to 95 % of tumor volume (D_95_), the maximum dose and the volume receiving 105 % of prescribed dose (V_105_) were evaluated (Table 1). Dose requirements for target volume and dose constraints for the OARs were established according to international dosimetric protocols, such as The Quantitative Analysis of Normal Tissue Effects in the Clinic (QUANTEC) and RTOG [25,26].

**Table 1:** Dose constraints and dose requirements for carrying out the treatment plan [25,26].

Target volume	Dose requirements	
**CTV**	D_95_ > 95 %	>47.50Gy
	D_max_ < 110 %	<55Gy
	V_105_ < 5 %	V_52.50Gy_ < 5 %
**IB**^a^	D_95_ > 95 %	>62.70Gy
	D_max_ < 110 %	<72.60Gy
	V_105_ < 5 %	V_69.30Gy_ < 5 %
**Organs at risk**	**Dose constraints**	

**HEART**	D_mean_ < 4Gy	
	V_25_ < 10 %	
	D_max_ = ALARA	
**IP. LUNG**	V_20_ < 30 %	
	D_mean_ < 15Gy	
**C. LUNG**	V_5_ < 15 %	
	D_mean_ = ALARA	
	D_max_ = ALARA	
**IP. H**	D_max_ < 42–45Gy	

**Abbreviations:** CTV = clinical target volume, IB = integrated boost, IP. LUNG = ipsilateral lung, C. LUNG = contralateral lung, IP. H = ipsilateral humerus, ALARA = As Low As Reasonably Achievable, D_95_ = 95 % of prescribed dose, D_max_ = Maximum dose, V_105_ (25, 20, 5) = % of volume receiving over 105 (25, 20, 5)% of prescribed dose, D_mean_ = Mean dose.

a Integrated boost was evaluated for equivalent dose in 2 Gy/fraction (EQD_2_).

Plans for conformal therapy were made with Collapsed Cone algorithm using 2 standard tangential fields for breast irradiation, 2 or 3 fields for lymph nodes (1 or 2 anterior fields and 1 posterior field) and 4 or 5 fields with 30° between each field for boost irradiation. In addition, plan optimization was performed manually, through Field-in-Field (FIF) technique, for maximum dose control and OARs dose reduction. A single isocenter was used and set between the breast and lymph nodes volumes, if irradiated. If the lymph nodes were not irradiated, the isocenter was placed in the center of breast volume. The established dose was prescribed using different reference points, one for each target volume: CTV (breast and lymph nodes) and boost.

IMRT and VMAT plans were created with Monte Carlo-based algorithms for inverse planning, using 6 fields for IMRT plans whereas for VMAT plans 2 anterior and 2 posterior semiarcs were used. Cost functions were employed for plan optimization. The isocenter and the prescribed dose were set in the center of CTV. Furthermore, the quality of treatment plans was achieved by evaluating the following normalization parameters: D_98 %_, D_95 %_, D_2%_, D_mean_, D_max_ and V_105 %_.

### 2.6 Statistical analysis

Body mass index was calculated for all patients to assess the correlation with setup errors. The association between two variables was evaluated by the Pearson correlation coefficient (r, which should have a value of ∼ ± 1 for a strong correlation) [27]. Weak, moderate and strong correlations were considered as follows: r between 0.000 and 0.400 was evaluated as weak correlation, r between 0.400 and 0.700 was associated with moderate correlation and r between 0.700 and 1 was considered a strong correlation.

Pearson correlation was also employed for BMI and breast size, which was another factor of interest in our study, as well as the association of breast size (this study evaluated the breast size as the contoured volume of the breast CTV) with setup errors to identify the occurrence of greater deviations from the original setup [27]. Another correlation was performed for treatment times (with and without EPID verification) and setup errors for both groups.

To evaluate the statistically significant results between the above-mentioned factors and setup errors as well as for dosimetric comparisons between the various radiotherapy techniques, a paired t-distribution (Student's t-distribution) was employed for p value calculations using GraphPad 10.0.3 [28]. The threshold for statistical significance was set at p < 0.05.

## 3 Results

### 3.1 Systematic and random errors according to the board inclination

Mean systematic and random errors were evaluated and presented in Table S1 from Supplementary materials for groups A and B for 3 standard directions of Cartesian coordinates (x = right/left, y = superior/inferior, z = anterior/posterior).

**Fig. 3:**
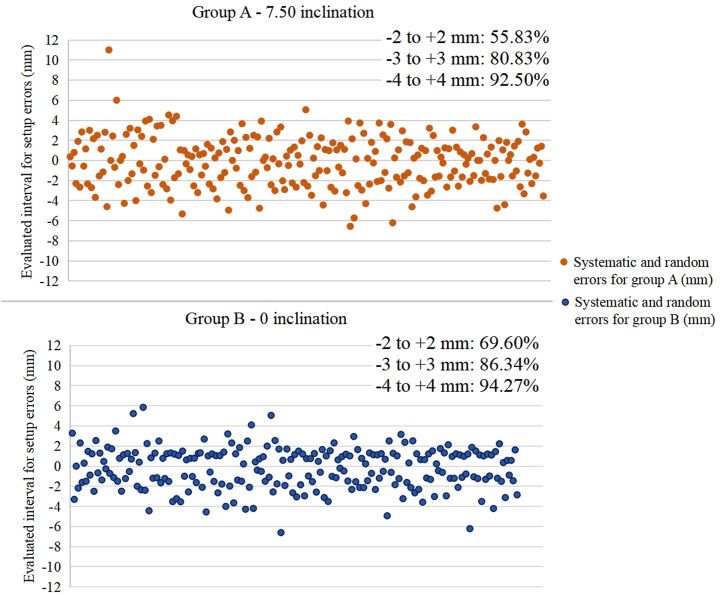
The percentage of setup errors located within the recommended limits (−2 and +2 mm) and beyond, for both patient groups. Setup errors were represented throughout the radiotherapy treatment for patients positioned on 7.5° inclination (group A) and on 0° inclination (group B).

Fig. 3 (A and B) shows the percentage of setup errors included in the range of interest (−2 to +2 mm) and two other intervals (−3 to +3 mm and −4 to +4 mm) to emphasize the differences between the two groups in terms of positioning errors.

### 3.2 PTV and OARs margins

PTV margins were calculated according to the setup errors reported in Table S1. The data resulting from margin calculations using van Herk et al.'s formula were over 5 mm for both groups, excepting the anterior direction in group B (4.77 mm). The values provided by Stroom et al.'s formula were around 5 mm for both groups, whereas the margins calculated with the ICRU 62 formula were below 5 mm for both groups (Table 2).

**Table 2:** CTV to PTV margins calculated with van Herk et al., Stroom et al. and ICRU 62 formula

M (mm) & M_PRV_ (mm)	Group A (7.5°)				Group B (0°)			
	PTV			OARs	PTV			OARs
	van Herk et al. [16]	Stroom et al. [18]	ICRU 62 [19]	McKenzie et al. [20]	van Herk et al. [16]	Stroom et al. [18]	ICRU 62 [19]	McKenzie et al. [20]
**right**	7.77	6.42	2.61	3.47	7.30	5.94	2.81	3.18
**left**	6.26	5.17	2.46	2.26	5.49	4.54	2.17	1.95
**superior**	6.66	5.55	2.72	2.12	5.61	4.67	2.29	1.80
**inferior**	8.23	6.83	3.31	2.76	6.63	5.46	2.59	2.48
**anterior**	6.01	5.11	2.63	1.61	4.77	3.98	1.97	1.47
**posterior**	8.08	6.81	3.56	2.04	9.24	7.85	4.28	2.01

**Abbreviation**: PTV = planning target volume, OARs = organs at risk, M = margins calculated for PTV creation, M_PRV_ = margins calculated for organs at risk.

For PRV calculation using McKensie et al. formula, the margins for group A and B were under 5 mm for all directions.

### 3.3 Correlations between BMI and setup errors

The BMI for both cohorts was calculated, showing no statistically significant difference between group A (mean BMI = 29.48 kg/m^2^, median BMI = 27.95 kg/m^2^) and B (mean BMI = 29.38 kg/m^2^, median BMI = 27.55 kg/m^2^), p = 0.478. The correlation between BMI and setup errors (presented in Table S1) is shown in Table 3. The results indicate a significant association between the variables in posterior direction for patients positioned on flat board (0° inclination) and a negative moderate correlation in the right, inferior (systematic errors) and superior (random errors) direction.

**Table 3:**
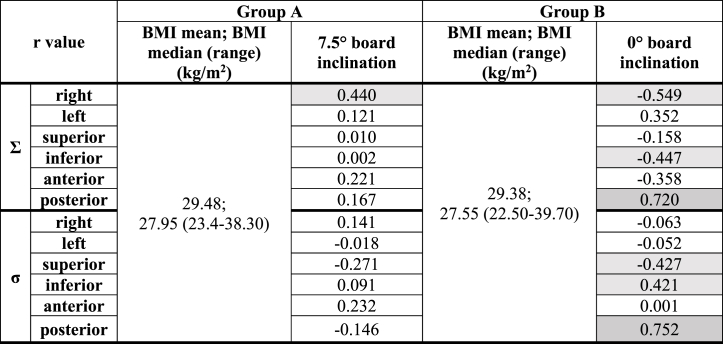
Pearson correlation coefficients (r value) between BMI and setup errors for both groups (moderate and strong correlations for r coefficient are highlighted in grey).

**Abbreviations**: Σ = systematic error, σ = random error, BMI = body mass index.

Mean systematic errors for tangential image parameters and their correlations with BMI and breast size were assessed. A negative moderate correlation (r = −0.533) was observed on CLD direction for 7.5° inclination between BMI and errors and a positive strong correlation along CCD (r = 0.786 BMI and r = 0.735 breast size) and CBESD (r = 0.786 breast size) for 0° inclination for BMI and breast size (Table 4).

**Table 4:**
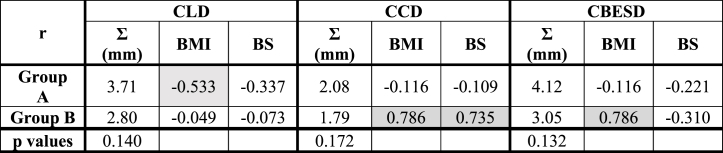
Mean systematic errors for tangential images parameters and r value calculation (moderate and strong correlations for r coefficient are highlighted in grey).

**Abbreviations**: BMI = body mass index, BS = breast size, r = Pearson correlation coefficient, CLD = central lung distance, CCD = central caudal distance, CBESD = central beam edge to skin distance.

The r value calculated for breast size and setup errors showed a moderate correlation along the right (−0.507) and left (0.503) directions (Table S2 from Supplementary materials), while in any other direction there was no notable correlation.

Moreover, a strong correlation was observed for both groups between breast size and BMI: 0.802 for group A and 0.799 for group B (p = 0.254).

### 3.4 Correlations between treatment times and setup errors

Moderate correlations (r = 0.504–0.614) were observed in the inferior direction for group A irrespective of the performance of image verification (Table S3 from Supplementary materials). Positive moderate correlation was observed in the right direction for random errors and negative moderate correlation in left direction for systematic errors for patients in group B. In addition, positive moderate (r = 0.585–0.618) and strong correlations (r = 0.717) were noticed in posterior direction in group B with and without EPID acquisition.

### 3.5 The effect of radiotherapy techniques on setup errors

**Fig. 4:**
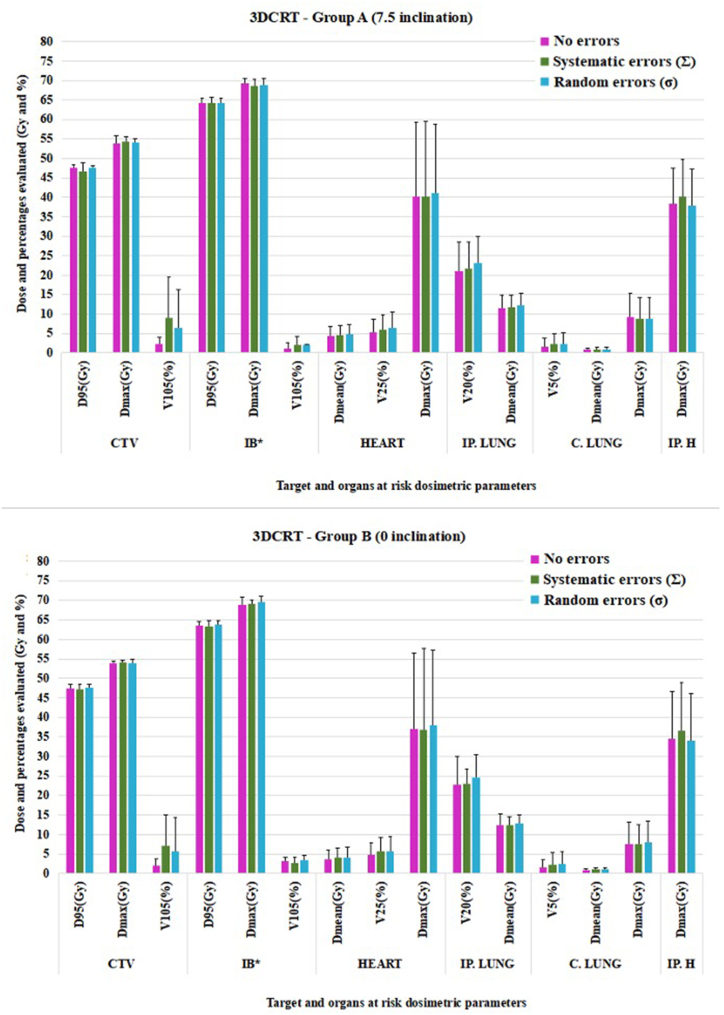
Mean target and OARs dosimetry evaluation under systematic and random errors for 3DCRT in groups A and B (CTV = clinical target volume, IB = integrated boost, IP. LUNG = ipsilateral lung, C. LUNG = contralateral lung, IP. H = ipsilateral humerus, D_95_ = 95 % of prescribed dose, D_max_ = Maximum dose, V_105_ (25, 20, 5) = % of volume receiving over 105 (25, 20, 5)% of prescribed dose, D_mean_ = Mean dose. ∗Integrated boost was evaluated for equivalent dose in 2 Gy/fraction (EQD_2_). The uncertainties in heart D_max_ were due to the fact the right- and left-sided breast cancer patients were evaluated together.

**Fig. 5:**
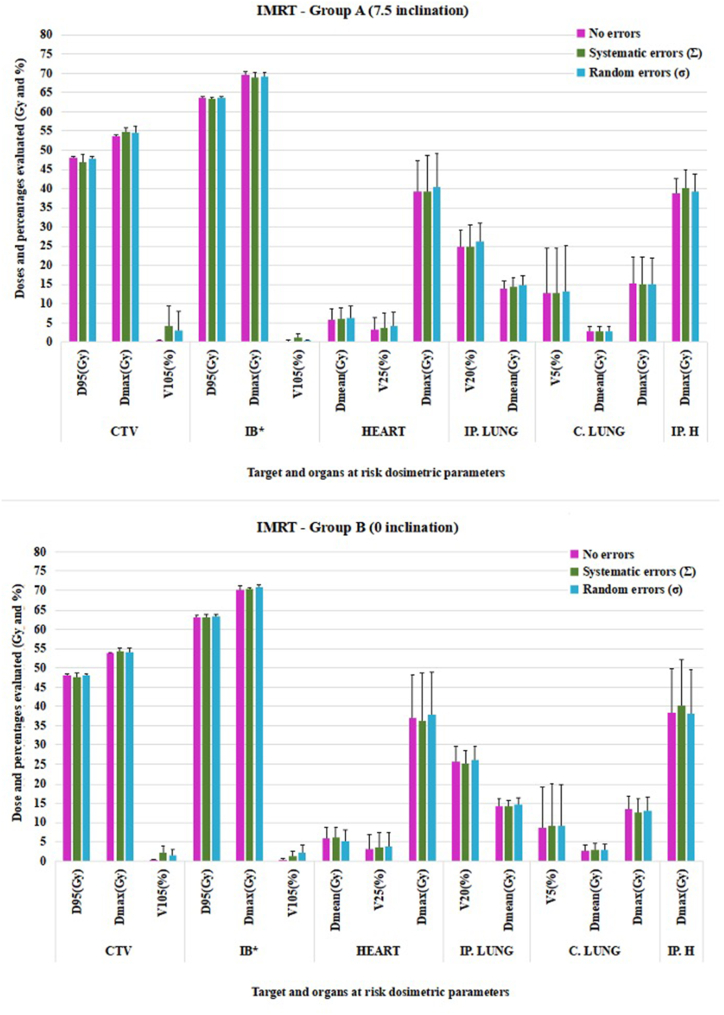
Mean target and OARs dosimetry evaluation under systematic and random errors for IMRT in groups A and B (CTV = clinical target volume, IB = integrated boost, IP. LUNG = ipsilateral lung, C. LUNG = contralateral lung, IP. H = ipsilateral humerus, D_95_ = 95 % of prescribed dose, D_max_ = Maximum dose, V_105_ (25, 20, 5) = % of volume receiving over 105 (25, 20, 5)% of prescribed dose, D_mean_ = Mean dose. ∗Integrated boost was evaluated for equivalent dose in 2 Gy/fraction (EQD_2_). The uncertainties in heart D_max_ were due to the fact the right- and left-sided breast cancer patients were evaluated together.

**Fig. 6:**
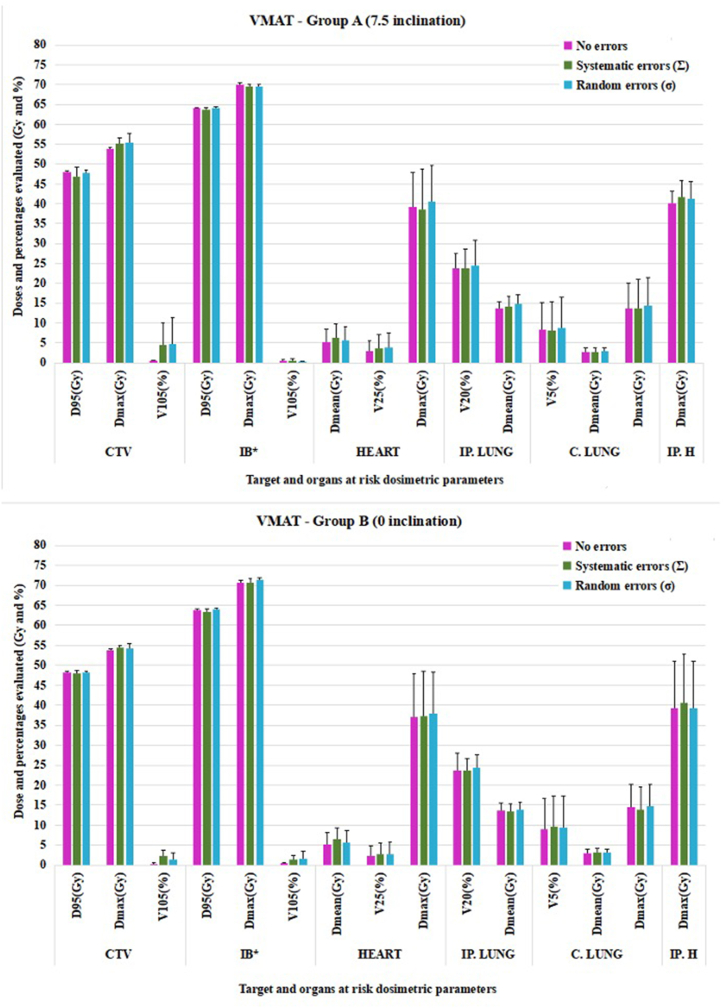
Mean target and OARs dosimetry evaluation under systematic and random errors for VMAT in groups A and B (CTV = clinical target volume, IB = integrated boost, IP. LUNG = ipsilateral lung, C. LUNG = contralateral lung, IP. H = ipsilateral humerus, D_95_ = 95 % of prescribed dose, D_max_ = Maximum dose, V_105_ (25, 20, 5) = % of volume receiving over 105 (25, 20, 5)% of prescribed dose, D_mean_ = Mean dose. ∗Integrated boost was evaluated for equivalent dose in 2 Gy/fraction (EQD_2_). The uncertainties in heart D_max_ were due to the fact the right- and left-sided breast cancer patients were evaluated together.

The simulation of setup errors on the treatment planning system for conformal and modulated techniques was assessed to evaluate the systematic and random errors. The obtained dosimetric results for the target and nearby organs at risk for both groups are presented in Fig. 4 (A and B), Fig. 5 (A and B) Fig. 6 (A and B) and Table S4 from Supplementary materials.

Among the assessed dosimetric parameters for the target volume, differences caused by induced errors were observed for D_95_ and V_105_. The CTV dose was minimally affected under systematic errors (∼93.60 % of prescribed dose for group A in all plans and 94.44 % for 3DCRT and >95 % for modulated plans in group B), while the dosimetric difference between the no-error and random error scenarios was approximately 0.15Gy for both groups.

For V_105_, the greatest difference between no-error versus induced errors was observed for conformal technique (∼5Gy for both groups), whereas for modulated techniques the difference was under 3Gy for group A and under 1.50Gy for group B.

According to our internal clinical protocol, the following organs at risk were evaluated: the heart, both lungs and ipsilateral humerus. Most dosimetric differences between the irradiation techniques were found in the contralateral lung. While in group A the V_5_ for 3DCRT plans was 1.53 %, for IMRT this value increased to 12.77 % whereas for VMAT to 8.25 %. Similar values for V_5_ were found in group B: 1.57 % for conformal plans, 8.56 % for IMRT and 9.05 % for VMAT.

To evaluate the statistical significance of the dosimetric differences between conformal and modulated techniques, p values were calculated and presented in the Supplementary Table S5 (3DCRT versus IMRT and IMRT versus VMAT for group B) and Table S6 (3DCRT versus IMRT and IMRT versus VMAT for group A).

Significant differences were observed in the contralateral lung for all evaluated parameters in all studied conditions (i.e. without errors and under the influence of systematic or random errors). In both groups, a statistically significant difference was reported for mean ipsilateral lung in all conditions, while for mean heart dose the difference between plans was observed in group B only.

Regarding target volume evaluation, differences were identified at the level of D_95_ and V_105_ parameters without the influence of setup errors for both groups. Also, between IMRT and VMAT plans, the only observable difference was for boost D_95_ in both groups without being influenced by setup errors.

## 4 Discussions

Accurate and reproducible patient positioning plays a critical role in a successful treatment delivery. Therefore, parameters related to treatment positioning, such as: hand cup height, arm height, headrest position, table height, distance from the mandible to the jugular cavity and the height of the immobilization system must be carefully recorded to avoid commencing radiotherapy with positioning inaccuracies that can transform into systematic errors.

The range of interest for systematic errors in our study was set between −2 mm and +2 mm according to the institutional protocol. Of the total occurrences of systematic and random errors for group A, 55.83 % fell within the interest range, the remaining 44.17 % being setup errors influenced by both the movement of internal organs and the relaxation of patients that caused sliding on the immobilization board. The indication for this assertion is given by the shifts along the inferior direction (2.80 mm for systematic errors and 1.76 mm for random errors), which were higher than for group B (2.34 mm and 1.17 mm) and also by the moderate correlation between time and setup errors for this direction (Σ: r = 0.504, σ: r = 0.602 with EPID verification and Σ: r = 0.564, σ: r = 0.614 without EPID acquisition). Deseyne et al. evaluated the sliding effect of two treatment positions (prone and supine) based on two immobilization systems, reaching the conclusion that the Crawl system (for prone position) had an advantage over the standard immobilization system (for supine position) as it prevents patients from sliding during treatment [29].

For group B, 69.60 % of the total occurrences of systematic and random errors were within the range of interest. Although the percentage of errors greater than 2 mm was found to be lower than for group A (positioned on 7.5° inclination), it is still significantly increased (30.40 %). The larger errors observed in some cases are likely caused by the uncomfortable supine position for those patients; the fact that larger errors were observed along the posterior direction (2.73 mm in group B vs 2.53 mm in group A for systematic errors; 3.24 mm vs 2.53 mm for random errors) is an indication for patient relaxation during treatment. This consideration is strengthened by the strong correlation observed between treatment time without EPID acquisition and systematic errors, r = 0.717, and other moderate correlations detected for systematic and random errors with and without portal acquisition (r > 0.500) in posterior direction.

As an overall observation, the main error occurring on the inclined immobilization system (group A) is caused by patients sliding on the immobilization board despite the use of a bottom stopper accessory. For patients positioned on 0° inclination (group B), the most common systematic/random errors were along the posterior direction. This aspect is due to patients' relaxation during treatment, or the discomfort given by the immobilization board which consists of a solid material, resulting in the patients’ inability to comfortably maintain the treatment position.

Therefore, while the 0° inclination board helps to reduce setup errors by 13.80 % as compared to the 7.5° inclination board (errors included in - 2 mm and 2 mm range), due to back pain reported by patients at the end of the treatment, this plane seems to be more uncomfortable than the inclined one.

The occurrence of errors can also be influenced by the patients' weight in relation to their height. For this assessment, the body mass index was calculated and the correlation between those two variables (BMI and setup errors for each direction) was assessed. For group A, a moderate correlation between BMI and setup errors was found in the right direction (r = 0.440), meaning that the occurrence of setup errors was related to the patient's habitus. For group B, a strong correlation between BMI and systematic (r = 0.720), as well as random (r = 0.752) errors emerged on posterior (-z) direction. This observation suggests that patients with higher BMI present a difficulty in maintaining the position during the treatment sessions. This consideration is reinforced by the study of Zhao et al. which evaluated four different body types (normal weight, moderate, overweight and obese), through BMI calculation, in terms of setup error evaluation and showed higher setup errors for obese patients (>5 mm in all directions vs < 5 mm for other body types) due to isocenter shifts caused by the subcutaneous fat and weak self-control ability [30].

Although correlations between BMI and setup errors were also observed in our study, Batumalai et al. reported no significant association between either BMI or breast size and errors assessed with portal images. The only correlation was observed in case of CBCT with soft tissue registration acquisition for both variables (BMI and breast size) and along all three coordinates (r > 0.600) [15]. Nijsten et al. aimed to develop a model to predict DVH changes (using CTV parameters) due to setup errors, however no correlations with breast size were found [31]. In our study, the correlation between setup errors and breast size was observed in group B under systematic errors. Additionally, it was showed that not only breast size can influence the changes in setup errors, but also organ motion [32].

Regarding the impact of the type of setup error on treatment planning dosimetry in our study, the systematic errors for group A showed higher values than the random ones (Fig. 4, Fig. 5, Fig. 6 and Table S4 - Supplementary material). A significant difference was observed for D_95_ under no errors for both groups (p = 0.028 for A and p = 0.007 for B) and for V_105_ under no errors for both groups and under systematic errors for group B. The CTV coverage indicated subirradiation (<95 % of prescribed dose <47.50Gy) for both techniques: conformal (46.65Gy) and modulated radiotherapy (46.95Gy for IMRT and 46.90Gy for VMAT). At the same time, for group B, underdosed target volume under systematic errors was observed only for 3DCRT plans (47.22Gy - 94.44 %).

A statistical difference indicated by p values in both groups was showed in the integrated boost coverage. For group A, the coverage of IMRT plans showed 63.59Gy and 64.03Gy for VMAT (0.44Gy difference), with similar difference found in group B (0.53Gy). Although the same Monte Carlo algorithm and cost functions were used for both IMRT and VMAT plans, this difference could be due to the geometric arrangement of the beams.

In relation to the PTV margins calculated using the 3 formulas (by van Herk et al., Stroom et al. and ICRU 62), the following conclusions were drawn.

1. According to the setup errors reported in Table S1, the PTV must be created with margins greater than 6 mm (group A) and 4.77 mm (group B) to be included in 95 % of the prescribed dose (according to Van Herk's formula);
2. Group A presented values higher than 5 mm in all directions (resulting an underdosed PTV) and group B presented 50 % of values below 5 mm in the left, superior and anterior directions (according to the formula of Stroom et al.);
3. The PTV would be optimal according to the applied margins with ICRU 62 formula - all values were below 5 mm.

The values for PTV margins in our study were higher (>2 mm) than those reported by Alabedi et al. which also evaluated the margins applied to PTV through portal images (1.43 mm vertical, 2.35 mm lateral, 1.50 mm longitudinal) [33]. The study suggested that setup errors can be generated by several factors that should be taken into account for OAR protection (when PTV margins applied) such as: target area expansion due to swelling after irradiation, weight loss that can alter the delineation of the target and lung movement during CT simulation or portal image verification [33].

Regarding the dose to OARs, our study showed a negative moderate correlation between tangential parameters and BMI in group A for CLD (−0.533). This parameter deviation can influence pulmonary dosimetry, thus if the BMI is higher than the normal range (18.50–24.90 kg/m^2^), the deviation and the pulmonary complications can increase. A study conducted by Mukundan et al. evaluated the dosimetric implications of this parameter in two groups of patients (30 right-sided and 30 left-sided breast cancer patients) and showed a deviation of CLD parameter in the first group of approximately 5 mm and 10 mm in the second group [34]. Furthermore, CLD appeared to be linearly related to the lung dose, reporting a 5.8 % deviation for V_20_ for the first group and 7 % for the second group [34,35].

In our study, a difference of 0.73 % for lung V_20_ was observed in group A (20.94 % under no errors and 21.67 % under systematic errors) and 0.28 % for group B (22.81 % under no errors and 23.09 % under systematic errors) for 3DCRT plans, while the percentage difference for modulated plans showed lung sparing with a mean of 0.41 %. However, all modulated plans presented higher DVH metrics than the 3DCRT plans.

The CCD parameter showed a strong correlation with systematic errors for group B (0.786). The influence of this parameter on target volume indicated an underdosed or an overdosed target. Also, for this patient group, a moderate negative correlation between BMI and breast size was observed along the right direction (−0.507) and a positive one along the left direction (0.503). This means that patients with large breasts positioned on flat plane (0° inclination) can induce higher errors. No correlation between BMI and breast size was found for group A.

Organs at risk that showed statistical difference between 3DCRT and IMRT plans were the ipsilateral lung (mean dose under no errors and setup errors) and contralateral lung (showed significant difference for all evaluated constraints under setup errors and no errors). The lung is one of the most important organs nearby the target, thus, to avoid pulmonary complications caused by setup errors and unnecessary irradiation, modulated techniques lead to better organ sparing than conformal techniques. However, the low-dose volume (V_5_) differences between 3DCRT and IMRT/VMAT for contralateral lung poses a possible risk of secondary cancer. The study conducted by Zhang et al. evaluated 26 breast cancer patients who were treated using one of the following four treatment techniques: 3DCRT with wedge, tangential IMRT, 6 fields IMRT and 2 partial arc VMAT [36]. The study evaluated OARs dosimetry in terms of secondary cancer risk and concluded that the 6-field IMRT and VMAT presented the highest risk due to field geometry and increased number of monitor units (MU): 3DCRT 400–600 MU, tangential IMRT: 200–400 MU, 6 fields IMRT: 350–600 MU, VMAT: 350–700 MU. When compared to our study, the above results are similar in terms of V_5_ for both 3DCRT (1.5 % vs < 5 %) and IMRT/VMAT (8–13 % vs 5–10 %), which might suggest a similar clinical outcome.

For group B, a statistically significant difference was also observed for the mean heart dose. The heart is a critical organ when treating left-sided breast cancer. The average difference in mean heart dose between the right-sided and left-sided cancer patients was approximately 4Gy for 3DCRT plans, the lowest doses being observed in patients treated for right-sided cancers with modulated techniques (3Gy).

To protect the organs at risk in the vicinity of the target volume, some studies applied margins (1 cm LAD or 5 mm for the lung and heart) to consider breathing motion [12,37] whereas others calculated those margins based on the McKenzie et al. formula. A study conducted by Topolnjak et al. evaluated these margins for heart dose assessment with both online and offline image registration, showing comparable results with those reported by our study: margins between 1.30 and 2.30 mm for offline registration (1.61–3.47 mm group A and 1.47–3.18 mm group B in our study) and 1.40–2.10 mm for online [38]. The clinical relevance of these margins is given by the numerous studies that evaluated the threshold doses for the occurrence of heart and lung toxicity due to radiation exposure [39,40]. Darby et al. suggested that any additional Gy over mean heart dose can lead to cardiac complications, while Hanania et al. concluded that V_5_ greater than 65 % may lead to pulmonary complications [41,42].

Although the systematic and random errors led to dosimetric values that influence both target volume coverage (underdosage or overdosage compared to error-free dosimetry), as well as OARs dosimetry, the effect of these setup errors should be interpreted by taking into account the variation of each dosimetric parameter evaluated. Fig. 4, Fig. 5, Fig. 6 indicate the uncertainties of the reported dose-volume parameters.

Our study has certain limitations. The evaluated cohort consists of a small number of patients, which requires that the reported data to be interpreted in this context. Studies reporting small cohorts are effective in delivering a quick research result by describing an association between a certain intervention and its outcome; therefore, the data presented here should be used as a design for confirmatory studies with larger number of subjects [44]. Comparable to our study, other similar reports used small cohorts [14,15,17] with results that could be verified in larger studies before wider clinical implementation. Another shortcoming is the EPID imaging system used for position verification which offers no information about the soft tissue, such as heart position and breast tissue modifications during treatment. Furthermore, 2D portal image-based verifications are limited to the evaluation of translational errors and do not allow the assessment of rotational misses and deformations. PTV margins could be reduced with the aid of 3D information (with soft tissue registration or/and bony registration) provided by CBCT images. In addition, combining CBCT with other imaging systems (2D or SGRT) could lead to residual error identification and reduction [4]. While CBCT has seen wide implementation into clinics worldwide, there is still a significant number of radiotherapy centers that rely on EPID as their main verification system for patient positioning [33,34,43]. Therefore, our study's results apply only to clinics that use EPID-based patient position verification, and not to those that use CBCT/SGRT systems.

The findings of our study for clinical translation can be summarized as follows.

• Patients with high BMI showed higher setup errors in posterior direction. These could be reduced by using the inclined immobilization system, however other, independent factors might have additional impact on positioning errors. The literature showed that errors can be reduced by conducting more frequent position verifications.
• Patients with large breasts positioned on flat plane (0° inclination) can induce high systematic errors. As suggested by others, the frequency of image verification should be higher than weekly in order to reduce setup errors.
• Daily or more than once-a-week position verification leads to setup error reduction, especially systematic errors, which are mainly evaluated through the acquired portal/CBCT/SGRT images. Moreover, the increase in treatment time (“beam on” time, in which no images are acquired), leads to an increase in random error occurrence (patient movement on immobilization board/patient position changes as a function of breathing amplitude).
• A strong correlation was found between systematic errors and central caudal distance (CCD) for 0° inclination.
• Systematic errors can lead to more significant underdosage of the CTV in patients on the inclined board than in patients on flat plane.
• Comparing the doses received by the ipsilateral lung from conventional versus modulated techniques, the latter offers a better prospect to reduce setup error-induced dosimetric misses.
• The sparing of both lungs was superior with conformal radiotherapy compared to modulated techniques.
• The moderate but statistically significant differences between conformal and IMRT plans for target volume coverage indicate that modulated techniques are more robust to setup errors.

**Fig. 7:**
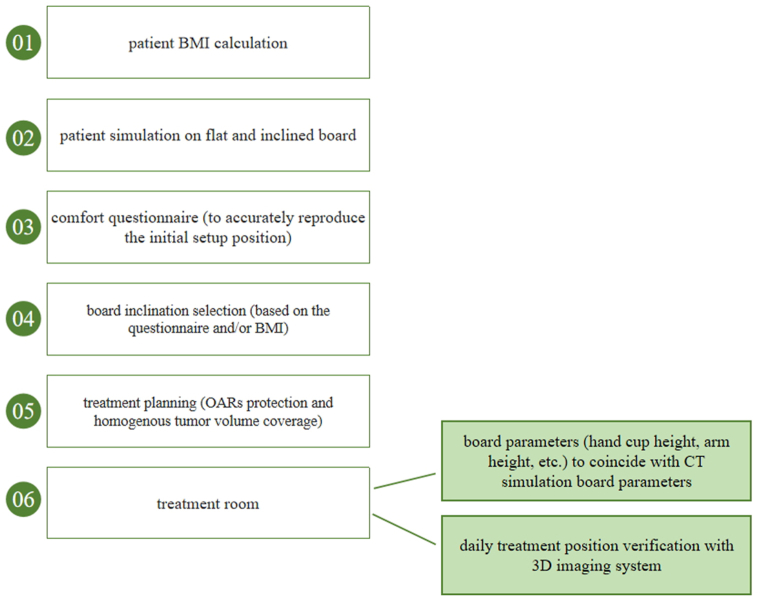
Schematic workflow (BMI = body mass index, OARs = organs at risk).

Practical implications for a clinical workflow that emerged from our study are presented in Fig. 7. As a future direction, the inclusion of a respiratory gating system would be effective in setup error reduction, along with OARs protection. Furthermore, using SGRT as a gating and treatment position verification system would be clinically efficient, reason why this technology is expected to become standard of care [45].

## 5 Conclusions

A more personalized treatment of breast cancer patients requires consideration of the patients’ anatomy when selecting an immobilization board as well as the inclination of the board. The body mass index and breast size are factors that can increase inconsistencies in patient positioning through systematic errors, as a function of board inclination. Additionally, the impact of setup errors on treatment techniques for different angulations of the immobilizing board can further influence the dosimetry of the target and of organs at risk.

## Ethics and consent declarations

Patient consent was waived due to the same reason as stated in the ethics committee approval.

## CRediT authorship contribution statement

**Ioana-Claudia Costin:** Writing – review & editing, Writing – original draft, Methodology, Investigation, Formal analysis, Conceptualization. **Loredana G. Marcu:** Writing – review & editing, Writing – original draft, Validation, Supervision, Conceptualization.

## Ethics committee approval

Ethical review and approval were waived for this study because it did not enroll patients for the said study. This work analyzed the data of patients that have been treated as per the normal medical protocol without any change induced by this study.

## Data availability statement author-disclosure

The data that support the findings of this study are available from the corresponding author upon reasonable request.

## Funding

This research received no funding. The article publication fee was supported by the University of Oradea.

## Declaration of competing interest

The authors declare that they have no known competing financial interests or personal relationships that could have appeared to influence the work reported in this paper
