# Supplementary material for: Impact of immobilization system angle, body mass index and breast size on breast radiotherapy accuracy using EPID-only setup
Source: Heliyon. 2025 Jan 22;11(3):e42176. doi: 10.1016/j.heliyon.2025.e42176 (PMC11830291; doi:10.1016/j.heliyon.2025.e42176)
Supplement: Multimedia component 1 [file mmc1.docx]

**Article Title:** Impact of immobilization system angle, body mass index and breast size on breast radiotherapy accuracy using EPID-only setup

**Journal name:** Heliyon

**Author names and affiliation:**

Ioana-Claudia Costin^1,2^, Loredana G. Marcu^3,4^

^1^ West University of Timisoara, Faculty of Physics, 300223, Timisoara, Romania

^2^ Bihor County Emergency Clinical Hospital, Oradea 410167, Romania

^3^ Faculty of Informatics & Science, University of Oradea, Oradea 410087, Romania

^4^ UniSA Allied Health & Human Performance, University of South Australia, Adelaide SA 5001, Australia

1. **mail address of the corresponding author:** [loredana.marcu@unisa.edu.au](mailto:loredana.marcu@unisa.edu.au) (Loredana G. Marcu)

Table S1. Mean systematic and random errors

| **Mean setup errors (range)** | | **Group A** | **Group B** |
| --- | --- | --- | --- |
|  |  | **7.5°** | **0°** |
| **Σ (mm)** | **right** | 2.95 (0.80 - 6.00) | 2.72 (1.15 - 5.85) |
|  | **left** | 2.18 (0.03 - 6.56) | 1.90 (0.43 - 3.32) |
|  | **superior** | 2.23 (0.21 - 5.05) | 1.88 (0.40 - 5.05) |
|  | **inferior** | 2.80 (1.17 - 5.73) | 2.34 (1.10 - 6.60) |
|  | **anterior** | 1.93 (0.10 - 11.00) | 1.58 (0.10 - 4.10) |
|  | **posterior** | 2.53 (0.19 - 4.60) | 2.79 (1.24 - 6.21) |
| **σ (mm)** | **right** | 0.74 (0.23 - 1.87) | 0.72 (0.23 - 1.23) |
|  | **left** | 1.15 (0.25 - 2.48) | 1.05 (0.27 - 1.92) |
|  | **superior** | 1.55 (0.23 - 3.43) | 1.30 (0.73 - 1.90) |
|  | **inferior** | 1.76 (0.27 - 3.67) | 1.12 (0.27 - 3.12) |
|  | **anterior** | 1.79 (0.25 - 4.53) | 1.17 (0.22 - 2.50) |
|  | **posterior** | 2.50 (0.34 - 4.95) | 3.24 (1.13 - 6.21) |
| **Abbreviations**: Σ = systematic error, σ = random error | | | |
