# Supplementary material for: Impact of immobilization system angle, body mass index and breast size on breast radiotherapy accuracy using EPID-only setup
Source: Heliyon. 2025 Jan 22;11(3):e42176. doi: 10.1016/j.heliyon.2025.e42176 (PMC11830291; doi:10.1016/j.heliyon.2025.e42176)
Supplement: Multimedia component 2 [file mmc2.docx]

**Article Title:** Impact of immobilization system angle, body mass index and breast size on breast radiotherapy accuracy using EPID-only setup

**Journal name:** Heliyon

**Author names and affiliation:**

Ioana-Claudia Costin^1,2^, Loredana G. Marcu^3,4^

^1^ West University of Timisoara, Faculty of Physics, 300223, Timisoara, Romania

^2^ Bihor County Emergency Clinical Hospital, Oradea 410167, Romania

^3^ Faculty of Informatics & Science, University of Oradea, Oradea 410087, Romania

^4^ UniSA Allied Health & Human Performance, University of South Australia, Adelaide SA 5001, Australia

1. **mail address of the corresponding author:** [loredana.marcu@unisa.edu.au](mailto:loredana.marcu@unisa.edu.au) (Loredana G. Marcu)

Table S2**.** Pearson correlation coefficients between breast size and setup errors (moderate correlation for r coefficient are highlighted in grey)

| **r** | **Group A** | | **Group B** | |
| --- | --- | --- | --- | --- |
|  | **Σ** | **σ** | **Σ** | **σ** |
| **right** | 0.282 | 0.361 | -0.507 | -0.091 |
| **left** | 0.168 | -0.135 | 0.503 | -0.096 |
| **superior** | -0.080 | -0.302 | -0.210 | -0.149 |
| **inferior** | 0.083 | 0.092 | -0.306 | 0.378 |
| **anterior** | -0.069 | 0.266 | -0.310 | 0.121 |
| **posterior** | 0.106 | -0.260 | 0.223 | 0.177 |
| **Abbreviations**: Σ = systematic error, σ = random error, r = Pearson correlation coefficient | | | | |
