# Supplementary material for: Impact of immobilization system angle, body mass index and breast size on breast radiotherapy accuracy using EPID-only setup
Source: Heliyon. 2025 Jan 22;11(3):e42176. doi: 10.1016/j.heliyon.2025.e42176 (PMC11830291; doi:10.1016/j.heliyon.2025.e42176)
Supplement: Multimedia component 3 [file mmc3.docx]

**Article Title:** Impact of immobilization system angle, body mass index and breast size on breast radiotherapy accuracy using EPID-only setup

**Journal name:** Heliyon

**Author names and affiliation:**

Ioana-Claudia Costin^1,2^, Loredana G. Marcu^3,4^

^1^ West University of Timisoara, Faculty of Physics, 300223, Timisoara, Romania

^2^ Bihor County Emergency Clinical Hospital, Oradea 410167, Romania

^3^ Faculty of Informatics & Science, University of Oradea, Oradea 410087, Romania

^4^ UniSA Allied Health & Human Performance, University of South Australia, Adelaide SA 5001, Australia

1. **mail address of the corresponding author:** [loredana.marcu@unisa.edu.au](mailto:loredana.marcu@unisa.edu.au) (Loredana G. Marcu)

Table S3. The correlation between treatment time with and without portal images verification (moderate and strong correlations for r coefficient are highlighted in grey)

| **r** | **right** | **left** | **superior** | **inferior** | **anterior** | **posterior** |
| --- | --- | --- | --- | --- | --- | --- |
| **Group A (7.5°)** | | | | | | |
| Treatment time with EPID verification | | | | | | |
| **Σ** | 0.124 | -0.372 | -0.267 | 0.504 | 0.231 | 0.113 |
| **σ** | 0.269 | 0.209 | 0.108 | 0.602 | 0.087 | -0.129 |
| Treatment time without EPID verification | | | | | | |
| **Σ** | 0.198 | -0.369 | -0.307 | 0.564 | 0.102 | 0.121 |
| **σ** | 0.266 | 0.122 | 0.030 | 0.614 | 0.059 | -0.093 |
| **Group B (0°)** | | | | | | |
| Treatment time with EPID verification | | | | | | |
| **Σ** | 0.222 | -0.454 | -0.042 | 0.262 | 0.226 | 0.618 |
| **σ** | 0.528 | -0.084 | 0.006 | -0.062 | -0.105 | 0.600 |
| Treatment time without EPID verification | | | | | | |
| **Σ** | 0.258 | -0.489 | -0.059 | 0.265 | 0.317 | 0.717 |
| **σ** | 0.573 | -0.045 | 0.037 | -0.072 | -0.197 | 0.585 |
| Abbreviations: Σ = systematic errors, σ = random errors, r = Pearson correlation coefficient | | | | | | |
