# Supplementary material for: Impact of immobilization system angle, body mass index and breast size on breast radiotherapy accuracy using EPID-only setup
Source: Heliyon. 2025 Jan 22;11(3):e42176. doi: 10.1016/j.heliyon.2025.e42176 (PMC11830291; doi:10.1016/j.heliyon.2025.e42176)
Supplement: Multimedia component 4 [file mmc4.docx]

**Article Title:** Impact of immobilization system angle, body mass index and breast size on breast radiotherapy accuracy using EPID-only setup

**Journal name:** Heliyon

**Author names and affiliation:**

Ioana-Claudia Costin^1,2^, Loredana G. Marcu^3,4^

^1^ West University of Timisoara, Faculty of Physics, 300223, Timisoara, Romania

^2^ Bihor County Emergency Clinical Hospital, Oradea 410167, Romania

^3^ Faculty of Informatics & Science, University of Oradea, Oradea 410087, Romania

^4^ UniSA Allied Health & Human Performance, University of South Australia, Adelaide SA 5001, Australia

1. **mail address of the corresponding author:** [loredana.marcu@unisa.edu.au](mailto:loredana.marcu@unisa.edu.au) (Loredana G. Marcu)

Table S4. Mean target and OARs dosimetry evaluation under systematic and random errors

| **Dosimetry evaluation** | | **3DCRT** | | | **IMRT** | | | **VMAT** | | |
| --- | --- | --- | --- | --- | --- | --- | --- | --- | --- | --- |
|  |  | **No errors** | **Σ** | **σ** | **No errors** | **Σ** | **σ** | **No errors** | **Σ** | **σ** |
| **Group A** | | | | | | | | | | |
| **CTV** | **D95(Gy)** | 47.57 | 46.65 | 47.63 | 47.98 | 46.95 | 47.81 | 48.08 | 46.90 | 47.78 |
|  | **Dmax(Gy)** | 53.74 | 54.35 | 54.09 | 53.58 | 54.73 | 54.57 | 53.73 | 55.15 | 55.36 |
|  | **V105(%)** | 2.27 | 8.93 | 6.36 | 0.19 | 4.11 | 3.09 | 0.21 | 4.60 | 4.81 |
| **IB*** | **D95(Gy)** | 64.26 | 64.23 | 64.32 | 63.59 | 63.37 | 63.54 | 64.03 | 63.63 | 64.10 |
|  | **Dmax(Gy)** | 69.21 | 68.60 | 68.80 | 69.57 | 69.00 | 69.23 | 69.89 | 69.56 | 69.52 |
|  | **V105(%)** | 1.13 | 1.93 | 2.04 | 0.24 | 1.03 | 0.35 | 0.22 | 0.48 | 0.04 |
| **HEART** | **Dmean(Gy)** | 4.41 | 4.51 | 4.70 | 5.84 | 5.91 | 6.12 | 5.29 | 6.41 | 5.63 |
|  | **V25(%)** | 5.30 | 5.87 | 6.32 | 3.31 | 3.76 | 4.15 | 2.92 | 3.67 | 3.93 |
|  | **Dmax(Gy)** | 40.09 | 40.26 | 41.01 | 39.15 | 39.13 | 40.47 | 39.27 | 38.63 | 40.55 |
| **IP. LUNG** | **V20(%)** | 20.94 | 21.67 | 23.07 | 24.89 | 24.84 | 26.19 | 23.85 | 23.88 | 24.51 |
|  | **Dmean(Gy)** | 11.53 | 11.79 | 12.31 | 13.99 | 14.28 | 14.71 | 13.81 | 14.12 | 14.74 |
| **C. LUNG** | **V5(%)** | 1.53 | 2.19 | 2.21 | 12.77 | 12.66 | 13.13 | 8.25 | 8.20 | 8.84 |
|  | **Dmean(Gy)** | 0.96 | 0.98 | 0.96 | 2.86 | 2.80 | 2.84 | 2.76 | 2.81 | 2.86 |
|  | **Dmax(Gy)** | 9.21 | 8.78 | 8.85 | 15.30 | 15.05 | 15.09 | 13.79 | 13.69 | 14.31 |
| **IP. H** | **Dmax(Gy)** | 38.38 | 40.26 | 37.91 | 38.62 | 40.12 | 39.21 | 40.18 | 41.80 | 41.29 |
| **Group B** | | | | | | | | | | |
| **PTV** | **D95(Gy)** | 47.48 | 47.22 | 47.65 | 48.05 | 47.66 | 48.01 | 48.25 | 47.95 | 48.16 |
|  | **Dmax(Gy)** | 53.77 | 54.12 | 53.96 | 53.78 | 54.36 | 54.11 | 53.85 | 54.33 | 54.17 |
|  | **V105(%)** | 2.13 | 7.20 | 5.64 | 0.28 | 2.25 | 1.43 | 0.29 | 2.20 | 1.33 |
| **IB*** | **D95(Gy)** | 63.62 | 63.32 | 63.81 | 63.25 | 63.15 | 63.45 | 63.78 | 63.44 | 63.93 |
|  | **Dmax(Gy)** | 68.84 | 68.99 | 69.45 | 70.16 | 70.29 | 70.91 | 70.71 | 70.75 | 71.34 |
|  | **V105(%)** | 3.20 | 2.69 | 3.34 | 0.38 | 1.33 | 2.09 | 0.20 | 1.44 | 1.71 |
| **HEART** | **Dmean(Gy)** | 3.70 | 4.05 | 4.12 | 5.84 | 5.99 | 5.13 | 5.16 | 6.43 | 5.53 |
|  | **V25(%)** | 4.74 | 5.74 | 5.81 | 3.19 | 3.63 | 3.70 | 2.33 | 2.77 | 2.79 |
|  | **Dmax(Gy)** | 36.96 | 36.84 | 37.98 | 36.95 | 36.25 | 37.97 | 37.13 | 37.34 | 37.91 |
| **IP. LUNG** | **V20(%)** | 22.81 | 23.09 | 24.70 | 25.60 | 25.17 | 26.06 | 23.62 | 23.71 | 24.25 |
|  | **Dmean(Gy)** | 12.29 | 12.50 | 12.88 | 14.28 | 14.11 | 14.65 | 13.69 | 13.32 | 13.87 |
| **C. LUNG** | **V5(%)** | 1.57 | 2.28 | 2.50 | 8.56 | 9.15 | 9.11 | 9.05 | 9.52 | 9.50 |
|  | **Dmean(Gy)** | 0.85 | 1.05 | 1.02 | 2.70 | 2.94 | 2.92 | 3.02 | 3.25 | 3.18 |
|  | **Dmax(Gy)** | 7.59 | 7.66 | 8.11 | 13.48 | 12.66 | 13.06 | 14.47 | 13.90 | 14.73 |
| **IP. H** | **Dmax(Gy)** | 34.57 | 36.63 | 34.09 | 38.26 | 40.08 | 38.03 | 39.24 | 40.61 | 39.32 |
| Abbreviations: CTV = clinical target volume, PTV = planning target volume, Σ = systematic error, σ = random error, IB = integrated boost, IP. LUNG = ipsilateral lung, C. LUNG = contralateral lung, IP. H = ipsilateral humerus, D95 = 95% of prescribed dose, Dmax = Maximum dose, V105 (25, 20, 5) = % of volume receiving over 105 (25, 20, 5)% of prescribed dose, Dmean = Mean dose.  *Integrated boost was evaluated for equivalent dose in 2 Gy/fraction (EQD_2_) | | | | | | | | | | |
