# Supplementary material for: Impact of immobilization system angle, body mass index and breast size on breast radiotherapy accuracy using EPID-only setup
Source: Heliyon. 2025 Jan 22;11(3):e42176. doi: 10.1016/j.heliyon.2025.e42176 (PMC11830291; doi:10.1016/j.heliyon.2025.e42176)
Supplement: Multimedia component 5 [file mmc5.docx]

**Article Title:** Impact of immobilization system angle, body mass index and breast size on breast radiotherapy accuracy using EPID-only setup

**Journal name:** Heliyon

**Author names and affiliation:**

Ioana-Claudia Costin^1,2^, Loredana G. Marcu^3,4^

^1^ West University of Timisoara, Faculty of Physics, 300223, Timisoara, Romania

^2^ Bihor County Emergency Clinical Hospital, Oradea 410167, Romania

^3^ Faculty of Informatics & Science, University of Oradea, Oradea 410087, Romania

^4^ UniSA Allied Health & Human Performance, University of South Australia, Adelaide SA 5001, Australia

1. **mail address of the corresponding author:** [loredana.marcu@unisa.edu.au](mailto:loredana.marcu@unisa.edu.au) (Loredana G. Marcu)

Table S5. P value calculation for group B (0° inclination) - statistically significant values highlighted in orange.

| **Target and OARs** | **Dose evaluation** | **3DCRT vs IMRT** | | | **IMRT vs VMAT** | | |
| --- | --- | --- | --- | --- | --- | --- | --- |
|  |  | **No errors** | **Σ** | **σ** | **No errors** | **Σ** | **σ** |
| **CTV** | **D95(Gy)** | 0.007 | 0.203 | 0.059 | 0.069 | 0.315 | 0.327 |
|  | **Dmax(Gy)** | 0.888 | 0.822 | 0.838 | 0.952 | 0.986 | 0.966 |
|  | **V105(%)** | <0.001 | 0.023 | 0.085 | 0.819 | 0.900 | 0.713 |
| **IB** | **D95(Gy)** | 0.490 | 0.829 | 0.482 | 0.048 | 0.574 | 0.083 |
|  | **Dmax(Gy)** | 0.245 | 0.045 | 0.094 | 0.332 | 0.335 | 0.204 |
|  | **V105(%)** | 0.584 | 0.479 | 0.351 | 0.353 | 0.881 | 0.762 |
| **HEART** | **Dmean(Gy)** | 0.007 | 0.017 | 0.018 | 0.718 | 0.621 | 0.666 |
|  | **V25(%)** | 0.177 | 0.082 | 0.101 | 0.374 | 0.379 | 0.368 |
|  | **Dmax(Gy)** | 0.999 | 0.907 | 0.999 | 0.954 | 0.746 | 0.983 |
| **IP. LUNG** | **V20(%)** | 0.105 | 0.120 | 0.461 | 0.114 | 0.191 | 0.195 |
|  | **Dmean(Gy)** | 0.013 | 0.015 | 0.015 | 0.308 | 0.155 | 0.133 |
| **C. LUNG** | **V5(%)** | 0.010 | 0.014 | 0.016 | 0.884 | 0.915 | 0.910 |
|  | **Dmean(Gy)** | <0.001 | <0.001 | <0.001 | 0.443 | 0.515 | 0.526 |
|  | **Dmax(Gy)** | 0.004 | 0.007 | 0.009 | 0.668 | 0.591 | 0.461 |
| **IP. H** | **Dmax(Gy)** | 0.379 | 0.424 | 0.345 | 0.791 | 0.892 | 0.730 |
| Abbreviations: CTV = clinical target volume, Σ = systematic error, σ = random error, IB = integrated boost, IP. LUNG = ipsilateral lung, C. LUNG = contralateral lung, IP. H = ipsilateral humerus, D95 = 95% of prescribed dose, Dmax = Maximum dose, V105 (25, 20, 5) = % of volume receiving over 105 (25, 20, 5)% of prescribed dose, Dmean = Mean dose. | | | | | | | |
