# Supplementary material for: Impact of immobilization system angle, body mass index and breast size on breast radiotherapy accuracy using EPID-only setup
Source: Heliyon. 2025 Jan 22;11(3):e42176. doi: 10.1016/j.heliyon.2025.e42176 (PMC11830291; doi:10.1016/j.heliyon.2025.e42176)
Supplement: Multimedia component 6 [file mmc6.docx]

**Article Title:** Impact of immobilization system angle, body mass index and breast size on breast radiotherapy accuracy using EPID-only setup

**Journal name:** Heliyon

**Author names and affiliation:**

Ioana-Claudia Costin^1,2^, Loredana G. Marcu^3,4^

^1^ West University of Timisoara, Faculty of Physics, 300223, Timisoara, Romania

^2^ Bihor County Emergency Clinical Hospital, Oradea 410167, Romania

^3^ Faculty of Informatics & Science, University of Oradea, Oradea 410087, Romania

^4^ UniSA Allied Health & Human Performance, University of South Australia, Adelaide SA 5001, Australia

1. **mail address of the corresponding author:** [loredana.marcu@unisa.edu.au](mailto:loredana.marcu@unisa.edu.au) (Loredana G. Marcu)

Table S6. P value calculation for group A (7.5° inclination) - statistically significant values highlighted in orange

| **Target and OARs** | **Dose evaluation** | **3DCRT vs IMRT** | | | **IMRT vs VMAT** | | |
| --- | --- | --- | --- | --- | --- | --- | --- |
|  |  | **No errors** | **Σ** | **σ** | **No errors** | **Σ** | **σ** |
| **CTV** | **D95(Gy)** | 0.028 | 0.620 | 0.258 | 0.316 | 0.927 | 0.857 |
|  | **Dmax(Gy)** | 0.996 | 0.860 | 0.767 | 0.934 | 0.823 | 0.828 |
|  | **V105(%)** | <0.001 | 0.121 | 0.222 | 0.882 | 0.998 | 0.553 |
| **IB** | **D95(Gy)** | 0.326 | 0.258 | 0.248 | 0.038 | 0.496 | 0.051 |
|  | **Dmax(Gy)** | 0.649 | 0.679 | 0.646 | 0.527 | 0.342 | 0.602 |
|  | **V105(%)** | 0.528 | 0.670 | 0.238 | 0.863 | 0.777 | 0.316 |
| **HEART** | **Dmean(Gy)** | 0.099 | 0.129 | 0.130 | 0.641 | 0.627 | 0.630 |
|  | **V25(%)** | 0.083 | 0.115 | 0.110 | 0.679 | 0.944 | 0.852 |
|  | **Dmax(Gy)** | 0.841 | 0.815 | 0.904 | 0.964 | 0.872 | 0.978 |
| **IP. LUNG** | **V20(%)** | 0.048 | 0.121 | 0.099 | 0.403 | 0.566 | 0.345 |
|  | **Dmean(Gy)** | 0.007 | 0.010 | 0.010 | 0.742 | 0.840 | 0.975 |
| **C. LUNG** | **V5(%)** | <0.001 | 0.001 | 0.001 | 0.147 | 0.156 | 0.186 |
|  | **Dmean(Gy)** | <0.001 | <0.001 | <0.001 | 0.750 | 0.961 | 0.970 |
|  | **Dmax(Gy)** | 0.005 | 0.004 | 0.003 | 0.476 | 0.554 | 0.728 |
| **IP. H** | **Dmax(Gy)** | 0.923 | 0.959 | 0.618 | 0.212 | 0.288 | 0.199 |
| Abbreviations: CTV = clinical target volume, Σ = systematic error, σ = random error, IB = integrated boost, IP. LUNG = ipsilateral lung, C. LUNG = contralateral lung, IP. H = ipsilateral humerus, D95 = 95% of prescribed dose, Dmax = Maximum dose, V105 (25, 20, 5) = % of volume receiving over 105 (25, 20, 5)% of prescribed dose, Dmean = Mean dose. | | | | | | | |
